# Seasons of life

**DOI:** 10.1017/S1478951524001809

**Published:** 2025-01-14

**Authors:** Antonio Yaghy

**Affiliations:** Department of Ophthalmology and Visual Sciences, University of Massachusetts Chan Medical School, Worcester, MA, USA

Her name was Grace, and as I would come to learn, it was a name that perfectly captured the essence of her being. As a medical student on my palliative care rotation, I had the privilege of being part of Grace’s journey through the seasons of her final months. She taught me profound truths about dignity, humanity, and the cyclical nature of life and care.

## Spring: The awakening of awareness

I first met Grace on a crisp spring morning, the air still holding a hint of winter’s chill. As I walked into her hospital room, the gentle sunlight filtering through the window bathed her face in a soft glow. The trees outside were just beginning to bud, their delicate green leaves unfurling hesitantly, as if testing the warmth of the new season. In a way, they mirrored the awakening awareness in Grace’s eyes as she grappled with the weight of her recent diagnosis of metastatic skin melanoma.

She turned to me as I approached her bedside, her gaze clear and steady despite the gravity of her situation. “So, young doctor,” she said, her voice soft but unwavering, “they tell me my time is limited. What do you think about that?”

Caught off guard by her directness, I hesitated, my mind searching for the right words. The sterile white walls of the hospital room seemed to close in on me, and the beeping of the monitors suddenly seemed deafening. “I … I’m not sure what to say, Grace,” I stammered, my voice barely above a whisper. “I’m sorry you’re facing this.”

She chuckled gently, the sound like a soothing balm to my frayed nerves. “Don’t be sorry, dear. Be honest. That’s all any of us can ask for in this life, isn’t it?” Her eyes, a striking shade of blue, held a depth of wisdom that belied her age, teaching me that honesty delivered with compassion is the foundation of dignified care.

As spring progressed, so did our conversations, each visit to Grace’s room became a treasure, a chance to delve deeper into the mysteries of life and death, of hope and acceptance. We talked about her life, her loves, her regrets, and her dreams. She shared stories of her youth, of the adventures she had embarked upon and the lessons she had learned along the way.

## Summer: The flourishing of connections

As summer arrived, I witnessed a similar blossoming in Grace’s room. Warm weather drew family members and old friends began to visit more frequently, filling the space with laughter, tears, and countless shared memories, creating an atmosphere that was at once joyful and bittersweet.

One afternoon, I found Grace surrounded by photo albums, their pages worn and slightly faded, but still holding the precious moments of her life. Her eyes twinkled with joy as she beckoned me closer.

“Ah, perfect timing!” she exclaimed, her voice filled with excitement. “Come, sit with me. I want to show you something.”

I pulled up a chair beside her bed. For the next hour, Grace guided me through the seasons of her life captured in those images, each one a window into a different time, a different story.

She spoke of love, of the man she had married and the life they had built together. She told me about their adventures, the trips they had taken, and the challenges they had faced hand in hand. Her voice grew soft as she recounted the small moments that had meant the most – a quiet sunrise shared on a mountaintop, a spontaneous dance in the kitchen, a tender embrace after a long day. As she turned the pages, I saw a reflection of the vibrant spirit that still shone within her, undimmed by the passage of time or the trials of illness.

“You know,” she mused, her fingers tracing the edge of a particularly cherished photograph, “I always thought I’d be afraid at the end. But looking at all this, all I feel is gratitude. Isn’t that something?”

I left Grace’s room that day with a heart echoing her profound sense of gratitude and a mind awakened to the beauty of a life well-lived. Her words had illuminated a truth I had yet to fully grasp: that in our final moments, it’s not fear that defines us, but the sum of our experiences and the love we’ve shared.

## Autumn: The harvest of reflection

As the leaves outside began to turn, painting the world in warm hues of amber, crimson, and gold, Grace entered a period of deep reflection. The change of seasons seemed to mirror the introspective journey she had embarked upon, and our conversations took on a more philosophical tone, diving into the profound questions that often arise when one is confronted with the finite nature of life.

“What do you think happens after, doctor?” she asked one day, her gaze fixed on the golden leaves outside her window. The question hung in the air between us, weighted with the gravity of its implications.

I took a moment to consider my response, knowing that my words carried a significance beyond the mere exchange of ideas. “I’m not sure, Grace,” I admitted, my voice soft with the humility of uncertainty. “What do you think?”

She smiled then, a gentle curve of her lips that seemed to hold a wisdom far beyond her years. “I think it’s like autumn,” she said. “We let go, but we also leave something behind. Our stories, our impact on others. That’s our harvest, isn’t it?”

Her words resonated deeply, painting a vivid picture of life’s seasons and the legacy we cultivate. As we continued our conversations, capturing her memories and reflections, I watched Grace embrace this autumnal phase of her life with a quiet dignity. Like leaves turning to brilliant hues before they fall, her stories took on new depth and meaning.

The process of articulating her life’s journey seemed to bring Grace a profound sense of peace. She spoke of the seeds she’d planted throughout her years – relationships nurtured, kindnesses shared, lessons imparted. These, she realized, were the fruits of her lifetime, ready to nourish those she would leave behind.

As the leaves outside continued their graceful descent, mirroring the gentle transition Grace herself was undergoing, I understood that her legacy was already taking root. It lived in the warmth of her smile, the wisdom of her words, and the lives she had touched with her indomitable spirit. Grace was right – this was her harvest, and it was bountiful beyond measure.

In helping Grace gather and share the golden leaves of her memories, I found myself learning a precious lesson about the seasons of life and the beauty of a graceful farewell. Her autumn was not an ending, but a transition – a time of letting go and leaving behind a legacy as enduring as the cycle of seasons itself.

## Winter: The embrace of peace

The final season of Grace’s journey came as softly as the first snow of winter, blanketing the world outside her window in a hushed and gentle silence. Even the hospital seemed to quieten around her room, as if respecting the sanctity of these last moments, the precious time that remained.

“It’s almost time, isn’t it?” Grace whispered one evening, her hand resting lightly in mine.

I nodded, feeling a lump in my throat, a weight of emotion that threatened to spill over. “Yes, I think so,” I managed, my voice soft and thick with the gravity of the moment. “Are you comfortable? Is there anything you need?”

She smiled then, her eyes clear and calm, reflecting a peace that seemed to emanate from the very depths of her being. “Just this,” she breathed, her words a gentle whisper in the stillness of the room. “Presence. Thank you for being here, for listening to an old woman’s stories.”

“Thank you for sharing them with me,” I replied, squeezing her hand gently, trying to convey through that simple gesture the profound impact she had had on my life, on my understanding of what it meant to be a healer. “You’ve taught me so much, Grace.”

In those quiet winter nights, as Grace peacefully took her last breaths surrounded by the loving presence of her family and friends, I learned perhaps the most important lesson of all: the power of simply being present, of bearing witness to the sacred transition from life to death, of honoring the inherent dignity in each moment until the very end.

As I sat by her bedside, holding space for her passage, I felt a deep sense of gratitude for the privilege of having known her, of having been a part of her journey. And in the stillness of those final hours, I understood that the essence of compassionate care lies not in the grand gestures or the heroic interventions, but in the quiet moments of connection, the simple act of being present with another human being during their last chapter.

## Conclusion: The eternal cycle of care

As I reflect on my time with Grace, I’m struck by how her journey mirrored the eternal cycle of the seasons, each phase bringing its own unique challenges and beauty. From the tentative spring of newfound awareness to the peaceful winter of acceptance and letting go, Grace taught me that end-of-life care is not about managing death, but about nurturing life in all its stages, right up until its natural conclusion.

In the end, Grace’s journey taught me that in honoring the dignity of others, we affirm our own humanity, our own connection to the great web of life that binds us all. And in this eternal cycle of care, we find the deepest expression of our compassion, the truest measure of our society’s values, and the most profound testament to the resilience and the beauty of the human spirit.

Her name was Grace, and true to that name, she taught me how to approach the end of life with dignity, with humanity, and yes, with grace. And in doing so, she left an indelible mark on my heart, a legacy of wisdom and compassion that will guide me through all the seasons of my own journey, both as a physician and as a fellow traveler on the path of life.

